# Qualitative alteration of peripheral motor system begins prior to appearance of typical sarcopenia syndrome in middle-aged rats

**DOI:** 10.3389/fnagi.2014.00296

**Published:** 2014-10-30

**Authors:** Tetsuro Tamaki, Maki Hirata, Yoshiyasu Uchiyama

**Affiliations:** ^1^Muscle Physiology and Cell Biology Unit, Tokai University School of MedicineIsehara, Japan; ^2^Division of Basic Clinical Science, Department of Regenerative Medicine, Tokai University School of MedicineIsehara, Japan; ^3^Division of Surgery, Department of Orthopedics, Tokai University School of MedicineIsehara, Japan

**Keywords:** muscle shortening-relaxation velocity, demyelination, axon loss, synaptic depression, neuromuscular junction, motor neuron pool, stretch-reflex

## Abstract

Qualitative changes in the peripheral motor system were examined using young, adult, middle-aged, and old-aged rats in order to assess before and after the appearance of sarcopenia symptoms. Significant loss of muscle mass and strength, and slow-type fiber grouping with a loss of innervated nerve fibers were used as typical markers of sarcopenia. Dynamic twitch and tetanus tension and evoked electromyogram (EEMG) were measured via electrical stimulation through the sciatic nerve under anesthesia using our force-distance transducer system before and after sciatectomy. Digital and analog data sampling was performed and shortening and relaxing velocity of serial twitches was calculated with tension force. Muscle tenderness in passive stretching was also measured as stretch absorption ability, associated with histological quantitation of muscle connective tissues. The results indicated the validity of the present model, in which old-aged rats clearly showed the typical signs of sarcopenia, specifically in the fast-type plantaris muscles, while the slow-type soleus showed relatively mild syndromes. These observations suggest the following qualitative alterations as the pathophysiological mechanism of sarcopenia: (1) reduction of shortening and relaxing velocity of twitch; (2) decline of muscle tenderness following an increase in the connective tissue component; (3) impaired recruitment of motor units (MUs) (sudden depression of tetanic force and EEMG) in higher stimulation frequencies over 50–60 Hz; and (4) easy fatigability in the neuromuscular junctions. These findings are likely to be closely related to significant losses in fast-type MUs, muscle strength and contraction velocity, which could be a causative factor in falls in the elderly. Importantly, some of these symptoms began in middle-aged rats that showed no other signs of sarcopenia. Thus, prevention should be started in middle age that could be retained relatively higher movement ability.

## Introduction

Sarcopenia is defined as the loss of muscle mass and strength that occurs with aging, leading to increased frailty, impaired mobility and loss of independence with general disability (Morley et al., 2001; Cruz-Jentoft et al., 2010; Mitchell et al., 2012). An increase in the associated loss of independence is a great burden for both affected individuals, and for caregivers and society (Lang et al., 2010); therefore, the precise mechanisms underpinning sarcopenia have been investigated vigorously for many years in order to prevent of these syndromes. Through numerous reports, it is likely that the loss of muscle mass is induced by an age-related decline in muscle fiber number and size, and this wholly depends on the age-related loss of spinal motor neurons (MNs), as defined by both animal and human data (Tomlinson and Irving, 1977; Edstrom and Larsson, 1987; Einsiedel and Luff, 1992; Deschenes, 2004). Consequently, reduced maximal muscle strength, power and rate of force development are induced, and this translates into impaired daily physical activities, such as walking, stair climbing and rising from a seated position, leading to an increase in fall risk (Thelen et al., 2000; Madigan and Lloyd, 2005; Maki and McIlroy, 2006; Zietz et al., 2011). To consider the physiological causes combined with falls, it has been reported that the ability to recover from a fall depends largely on maximum stepping speed (Thelen et al., 1997; Wojcik et al., 1999), except when there are specific pathological reasons via other sensory systems and/or the central nervous system. An age-related reduction in stepping speed could potentially be caused by a reduction in joint-movement velocity in each localized individual joint of the body, and this further depends on the strength and velocity of each muscle. Furthermore, the strength and velocity of the muscle depends a great deal on the state of motor unit (MU) recruitment, particularly the high-threshold fast-twitch MUs (FF type). In this regard, age-related quantitative reductions specific to Type II muscle fibers (Lexell, 1995; Larkin et al., 2003; Deschenes, 2004) are considered to be a supportive reason for accidental falls after a stumble. These quantitative declines following aging, associated with reduced Type II muscle fibers, have been widely reported. However, qualitative changes, such as the recruitment state of MUs, the contraction and relaxing velocities during twitch contraction, and the muscle tenderness have not been well-documented in relation to aging and sarcopenia. In addition, there has been little information to directly connect the quantitative and qualitative data in relation to before and after the appearance of sarcopenia.

In this study, therefore, we used young, adult, middle-aged, and old-aged rats in order to assess before and after the appearance of sarcopenia. On that basis, apparent muscle atrophy (decreased muscle mass) and appearance of slow-type fiber groupings with loss of innervated nerve axons were used as markers of sarcopenia, and we then measured muscle shortening and relaxation velocity during serial twitch contractions as the markers for myosin head oscillation and ATP-dependent Ca^++^ pump functions. To clarify the changes in peripheral motor nervous system, we also recorded evoked electromyogram (EEMG) and contractility in response to stimulation frequencies from 10 to 140 Hz associated with tension output of twitch and tetanus. These measurements were performed under *in situ* electrical stimulation via the sciatic nerve on fast-type plantaris (PLT) and slow-type soleus (SOL) muscles. Muscle tenderness of extensor digitorum longus (EDL) muscles during isokinetic stretching were also measured. The results indicated that functional deterioration preceded the appearance of sarcopenia syndromes, and further suggested that habitual recruitment of FF-MUs during middle age may be an important factor in preventing the earlier appearance of sarcopenia syndromes.

## Materials and methods

### Animals

Male Wistar rats aged 3 weeks were used as the Young group (*n* = 10), rats aged 12–17 weeks were used as the adult group (*n* = 28), rats aged 1.2–1.4 years were used as the middle-aged group (*n* = 11), and rats aged more than 2.5 years were used as the old-aged group (*n* = 17). Animals were housed in standard cages at a temperature of 23 ± 1°C and a 12-h light/dark cycle was used throughout the experiment. All experimental procedures were conducted in accordance with the Japanese Physiological Society Guide for the Care and Use of Laboratory Animals, and were approved by the Tokai University School of Medicine Committee on Animal Care and Use.

### Evaluation of sarcopenia

First evaluation of sarcopenia was performed based on the standard relationship between body and plantaris (PLT) muscle mass, as reported previously (Tamaki and Uchiyama, 1995). Basically, growth of the body and PLT muscle mass shows a proportional relationship equation curve, and hypertrophied and/or atrophied muscle is seen as a change in this equation curve (*n* = 528, aged 2–60 week-old, refer to **Figure 2**).

### Functional measurement

The twitch and tetanic tension outputs of the right plantaris (PLA) and left soleus (SOL) muscles were measured and compared among the adult (*n* = 27), middle-aged (*n* = 6), and old-aged (*n* = 11) groups. Measurements were performed *in situ* under inhalation anesthesia (Isoflurane; Abbot, Osaka, Japan), and body (rectal) temperature was maintained at 36 ± 1°C with a radiant heat light throughout the measurement. The reference muscles and sciatic nerves (about 15 mm) on both sides were carefully exposed, and tissues were coated with mineral oil to prevent tissue drying and to minimize electric-noise interference. A bipolar silver (Ag/Ag) electrode (inter-electrode distance: 2 mm) was placed under the sciatic nerve and a stainless steel hook was attached to the distal tendon of each reference muscles using silk ligature. The animal was transferred to a custom-made operating table that allowed stabilization of the head and limbs in a prone position using surgical tape. A stainless steel hook was attached to a force-distance transducer (FD-Pickup, TB-611T; Nihon Kohden, Tokyo, Japan) connected to a carrier amplifier (AP-621G; Nihon Kohden). A bipolar silver electrode (inter-electrode distance: 5/1 mm diameter) was also attached to the surface of the reference muscle in order to obtain an evoked electrical myogram (EEMG). Care was taken to avoid interference with the normal blood supply of the reference muscles. Twitches were then elicited using single pulse (1 ms duration, 0.5 Hz) electrical stimulation via the sciatic nerve, at a voltage above the threshold for maximum response (1.5–3.0 V). Serial twitch responses (10 times) were recorded on a personal computer after analog/digital (A/D) conversion (sampling rate was set at 5–10 kHz). Subsequently, the peak tetanic tension was determined using stimulation frequencies of 10, 20, 40, 60, 80, 100, 120, and 140 Hz at 10-s intervals. The frequency that produced the highest tetanic tension was considered to be the optimal stimulation for tetanus. All mechanical and electrical measurements were recorded on a Linearcorder (Mark VII, WR3101; Graphtec, Tokyo, Japan) as analog raw data. Digital data was stored on the personal computer using an A/D converter (MacADIOS II; GW Instruments, Somerville, MA) and was analyzed by SuperScript II software (GW Instruments). Importantly, in the present functional measurement, we used a force-distance transducer that was able to add a variable spring load at 0–500 g to maintain a linear and proportional relationship with 0–6.0 mm displacement. Using this force/distance transducer, we detected shortening and relaxation velocity during dynamic twitch contractions (10 twitches), and calculated these as 10% changes. Calibration of the force/distance transducer in the range used in twitch analysis is presented in Supplementary Figure S1.

### Measurement of muscle tenderness

**Figure 1:**
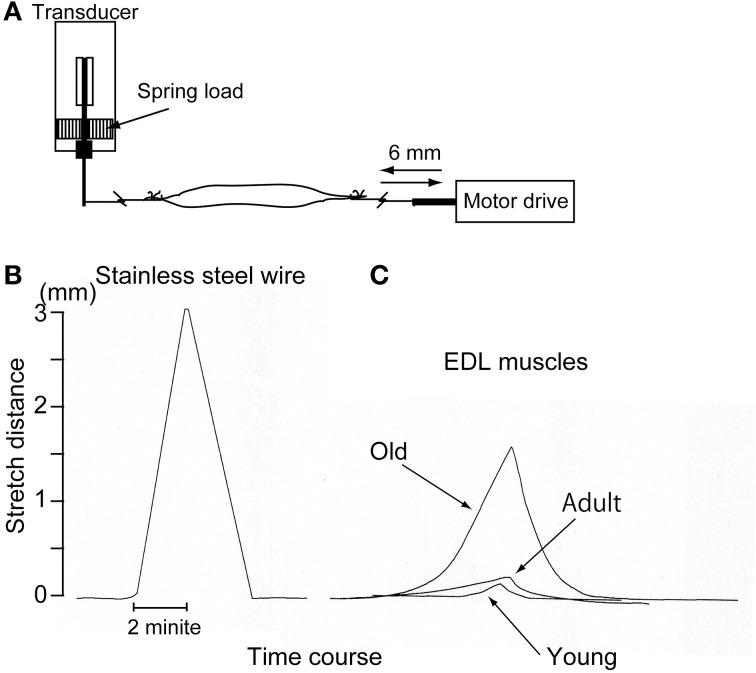
**Schematic drawing of the muscle tenderness measurement method (A) and representative raw data for stainless steel wire (B) and three types of muscle (C).** Muscle stretch/return speed was strictly regulated by electric motor drive micrometer at 1.5 mm/min. Stainless wire showed a symmetric response as it was a non-elastic substance **(B)**, but the muscles showed apparently different responses as they are elastic substances **(C)**. This measurement could be established by using a force/distance transducer, which would have the ability to vary with both the force and distance (see Supplementary Figure S1).

Muscle tenderness (isokinetic stretch-absorption) was also measured using the force/distance transducer above (TB-611T; Nihon Kohden, Figure 1A). In this measurement, EDL muscles were used because of their characteristics, as follows; (1) muscle-tendon junctions are easily detectable, and are on the distal and proximal ends; and (2) it is a multijoint muscle that spans the knee and ankle, thus receiving growth effects from crural bone length in the hindlimb. EDL muscles were obtained from the young (*n* = 4), adult (*n* = 5), and old-aged (*n* = 4) rats under overdose with sodium pentobarbital (60 mg/kg, i.p.). Stainless steel hooks were attached to both the distal and proximal ends. The proximal end was connected with the force/distance transducer, and the distal end was connected to the electric motor drive micrometer (EMDM). The muscle was then stretched in isokinetic mode (stable speed: 1.5 mm/min) using EMDM both absolutely (6 mm each) and relatively (14% of entire *in situ* EDL length), and return and displacement curves were recorded using Linearcorder (Graphtec). The outline of this method, and typical measurements are presented in Figure 1A. In this case, if the material had no elasticity, such as stainless steel wire, linear changes with absolute stretch length (3 mm) were recorded as symmetrical with the stretch-return phase (see Figure 1B). However, if the materials had elasticity and/or tenderness, stretch effects were absorbed and changes were reduced according to the stretch-absorbed capacity (see Figure 1C). Note that linearly increased spring load was equally added to the reference muscles during the 6-mm stretch and return phase. Values were presented as the absolute and relative muscle tenderness calculated as % absorption when stretched 6 mm each and/or 14% of the entire EDL muscle length *in situ*.

### Histochemical and immunohistochemical staining

Animals were anesthetized by overdose with sodium pentobarbital (60 mg/kg, i.p.), blood removal was performed through cardiac puncture, and plantaris (PLT) muscles were excised and weighed. In this analysis, adult (*n* = 6–10), middle-aged (*n* = 4–6), and old-aged (*n* = 4–10) rats were used. Muscles were then quick frozen in isopentane pre-cooled with liquid nitrogen, and were stored at −80°C until use. Subsequently, several 7-μm cross-sections were obtained. In order to detect the nerve fiber degeneration and/or decline in PLT muscle, localization of nerve fibers (axons) was detected using rabbit polyclonal anti-Neurofilament 200 (N-200, 1:1000, room temperature for 1 h; Sigma, Saint Louis, MO). Similarly, the presence of myelination was also detected by rabbit polyclonal anti-myelin basic protein (MBP; 1:200, room temperature for 2 h; Millipore, Billerica, MA). Rabbit polyclonal anti-collagen I (ab34714; 1:100, room temperature for 1 h; Abcam, Tokyo, Japan) was used to evaluate the increase in connective tissue content in the muscle following aging. In addition, myosin ATPase (mATPase) after acid preincubation (pH 4.3 and 4.6) was used to characterize the fiber type in the muscle. After staining of collagen and ATPase, we calculated the percentage of collagen Type I area, and the percentage distribution of Type-I, Type-IIa, and Type-IIb fibers per unit area using a Stereo-investigator system (mbf Bioscience; Micro Bright Field Inc., Williston, VT) and Photoshop (Adobe). Analysis was performed in 4–5 sections per sample, and 6–9 unit areas were selected in each section (refer to representative analysis in **Figure 11**).

### Statistical analysis

All data are expressed mean's ± *SE*. Differences between controls and experimental groups were determined using Dunnet *post-hoc* multi-comparison method, and Tukey–Kramer *post-hoc* multi-comparison was used for all individual differences. Differences between selected groups were determined by Student's *t*-test. Standard regression analysis and Pearson's product correlation procedures were used to determine the relationship between body and muscle mass, and calibration of the transducer. Differences were considered to be statistically significant at either the *P* < 0.05 (in the figures) or *P* < 0.01 (in the table) level.

## Results

### Confirmation of sarcopenia

**Figure 2:**
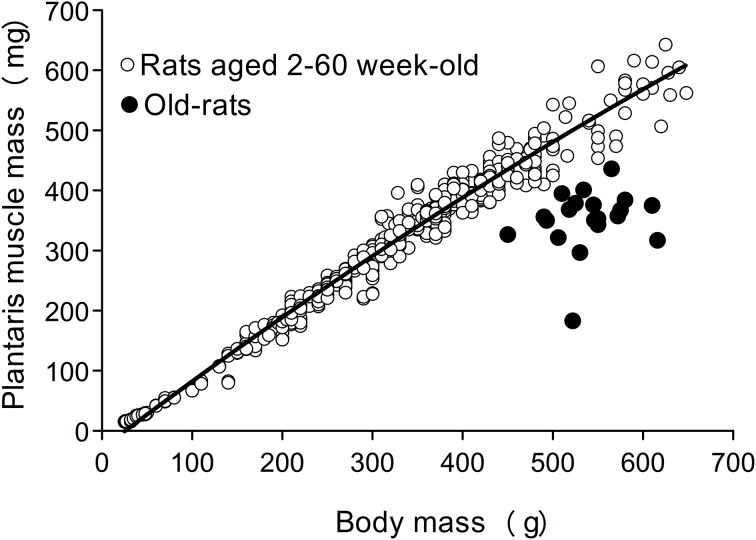
**The standard index for body and PLT muscle mass based on over 500 rats.** The equation for the correlation coefficient curve is y = −8E−10x^3^ + 6E−07x^2^ + 0.0009x − 0.0161, and *R*^2^ = 0.9735. Old-aged rats (closed circle) were clearly showed far below the standard curve, indicating muscle atrophy.

**Figure 3:**
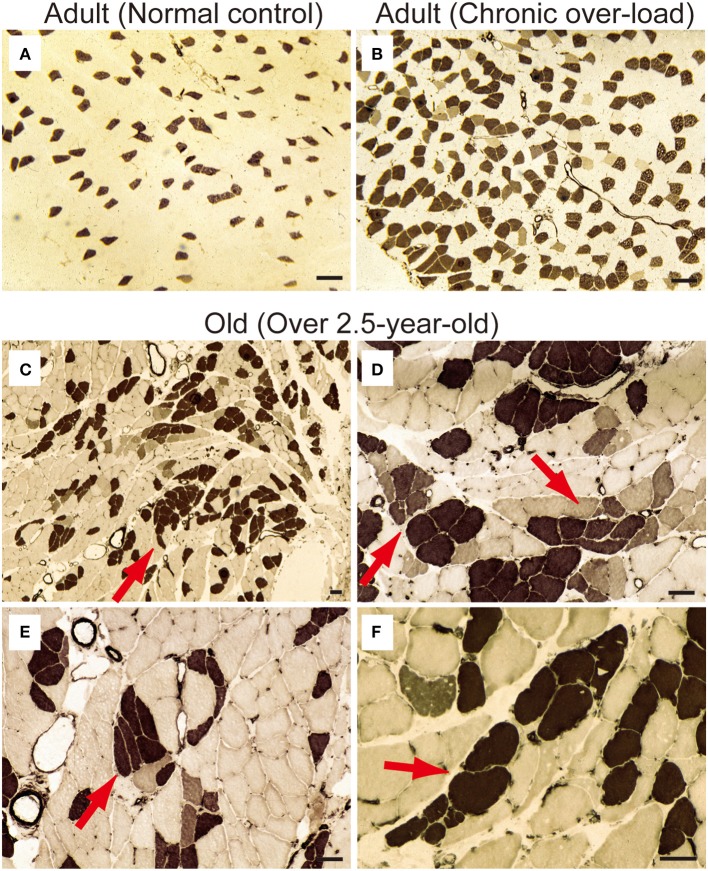
**ATPase staining for adult (A, 15-week-old) and old-aged (C–F, 136–139-week-old) rat PLT muscles.** ATPase staining was performed after incubation at pH 4.3; thus, Type I fibers were stained dark. Note that **(B)** is a comparative positive control for ATPase staining using compensatory hypertrophied PLT muscle after 6 weeks of surgical ablation of synergistic gastrocnemius and soleus muscles (16-week-old rats). This demonstrated that the shift to slow fibers occurred after chronic stretch stimulation. However, slow-type fiber grouping was evident in the old-aged PLT muscle **(C)**, suggesting remodeling of S-type motor units in the process of sarcopenia (red arrows in **C–F**). Panels **(D–F)** shows higher magnification of **(C)**. Bars in **(A,B)** = 100 μm, **(C–F)** = 50 μm.

**Figure 4:**
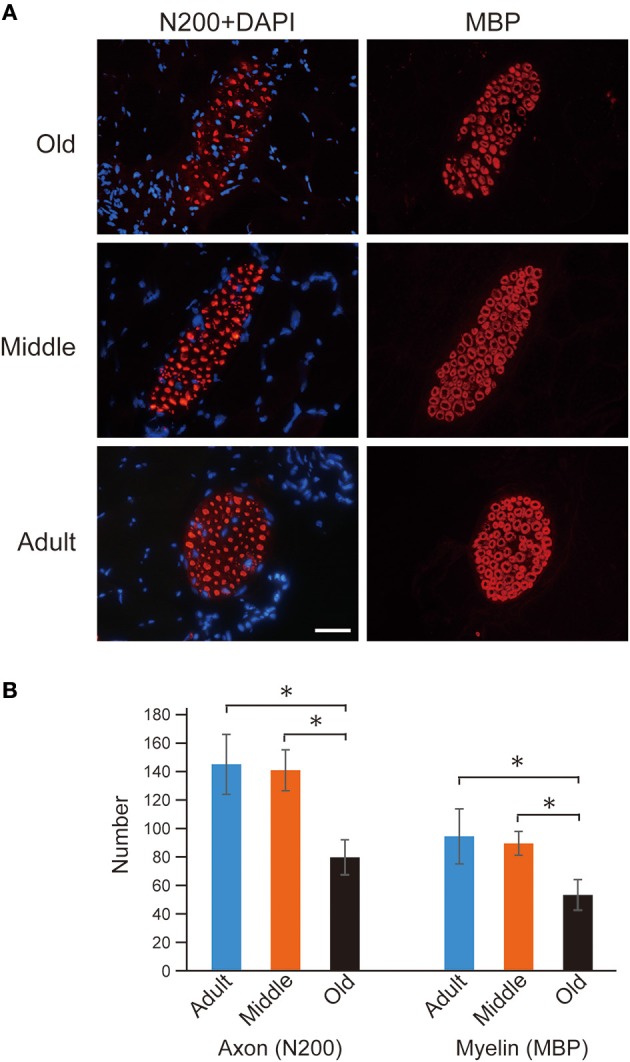
**Axon (N-200) and myelin (MBP) immunostaining for dominant nerve in PLT muscles (A), and their statistics (B).** Reactions were relatively sparse in the old-aged group when compared to middle-aged and adult rats **(A)**. The number of axons and myelin were 80 ± 12 and 53 ± 11 in old-aged, 141 ± 14 and 90 ± 8 in middle-aged, and 145 ± 21 and 95 ± 19 in adult, thus showing that significant decrease was occurred in the old-aged rats. Blue nuclear staining is DAPI. Bar = 50 μm and all photographs were at the same magnification. ^*^P < 0.05.

First, occurrence of sarcopenia in the muscles in old-aged rats was confirmed using standard indices for body and PLT muscle mass (Figure 2). This index was based on 528 rats, between 2 and 60 weeks of age, and weighing between 25 and 650 g. A very high correlation coefficient was observed between body and PLT muscle mass (*R*^2^ = 0.9735). However, the old-aged rats showed lower relative muscle masses from the established standard range, indicating that age-dependent muscle atrophy (sarcopenia) occurred. In addition, apparent increases in Type-I fibers with aggregated fiber-type grouping, which is a typical characteristic of sarcopenia (Lexell and Downham, 1991; Saini et al., 2009; Kung et al., 2014), was evident in the histochemical ATPase staining in PLT muscles in old-aged rats (Figures 3C–F), whereas uniform distribution was observed in adult rats (Figure 3A). Increases in Type-I fibers in basically fast PLT muscles of normal adult rats were also usually observed after 6 weeks of chronic stretch stimulation following surgical ablation of synergistic SOL and gastrocnemius muscles. However, in such cases, increases in Type-I fibers showed a relatively uniform trend, as was seen in normal controls (Figure 3B), as a result of physiological stimulation (chronic stretch). Therefore, the appearance of fiber-type groupings in the present old-aged rats was considered to be due to type S MU remodeling following degradation of type F (FF, FR) units, which was also a typical trend in sarcopenia. The above trends were also supported by the results of axons and myelin staining for the dominant nerves in PLT muscles in the old-aged, middle-aged, and adult rats (Figure 4A). Both types of staining were sparse in the old-aged rats, suggesting a relative decrease in the number of axons (red reactions of N200) and myelin (red reactions of MBP) in the old-aged rats, whereas the middle-aged and adult rats showed similar results. When there were calculated, the old-aged rats showed significantly lower values in the number of axons and myelin compared to both adult and middle-aged (Figure 4B). This also indicates age-dependent decreases in the number of MUs in the present old-aged rats. Note that apparent differences between the adult and middle-aged rats were not detected in this analysis.

### Assessment of muscle tension output

**Figure 5:**
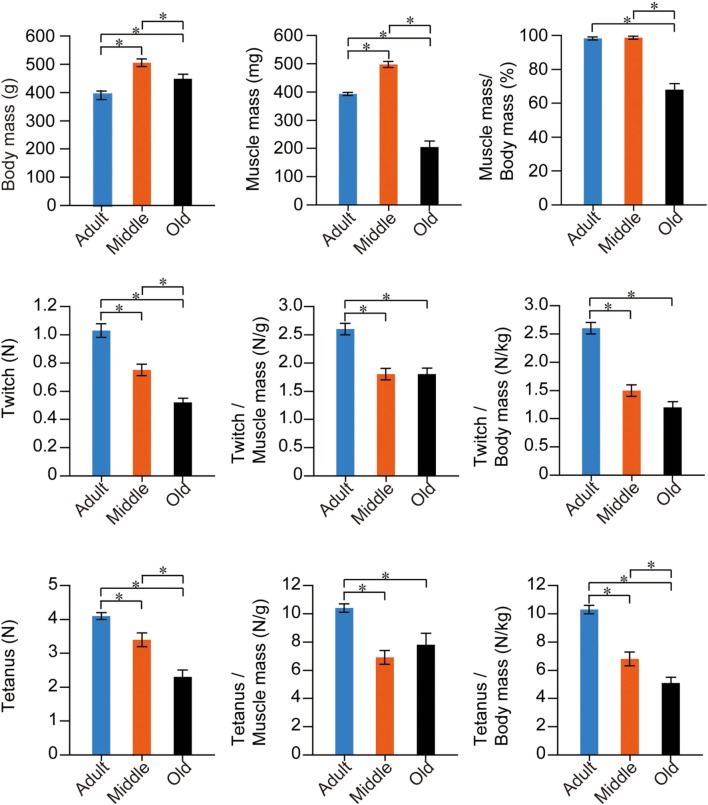
**Stats for PLT muscle functions in the three groups.** Muscle twitches and tetanus are expressed in terms of Newtons (N), and are evaluated as absolute and relative (per muscle and body mass) values. There were numerous significant differences among the three groups in all items in fast-type PLT muscles. ^*^p < 0.05

Using the above groups of rats, we measured *in situ* muscle contraction characteristics via electrical stimulation through the sciatic nerve and performed analyses based on body and muscle mass. The stats for PLT muscle functions are summarized in Figure 5. In this analysis, the older two groups showed significantly higher body mass than adults, with the highest levels seen in the middle-aged group. However, for PLT muscle mass, the same trend as for body mass was observed between the adult and middle-aged groups, but significantly lower values were observed in the old-aged group. Consequently, the muscle/body mass index (relative muscle mass) was largely the same in the adult and middle-aged groups, but the old-aged group showed significantly lower values, as was seen in Figure 2. For the functions, age-dependent significant decreases in absolute twitches and tetanus were observed among the three groups, and a similar trend was also seen in relative tensions/body mass. However, significant differences between the middle- and old-aged disappeared when they were compared by relative tension/muscle mass, while a significantly higher value was observed in the adult group. These results indicate two important points: (1) in the middle-aged rat PLT muscles, muscle atrophy was not seen, but functional decreases were already significant, and the assumed body-supportive effects were also significantly diminished; and (2) in the old-aged rat PLT muscle, three sarcopenia factors, functional deterioration and decreased body-supportive effects were apparent, but tension/muscle mass did not differ from the middle-aged group.

**Figure 6:**
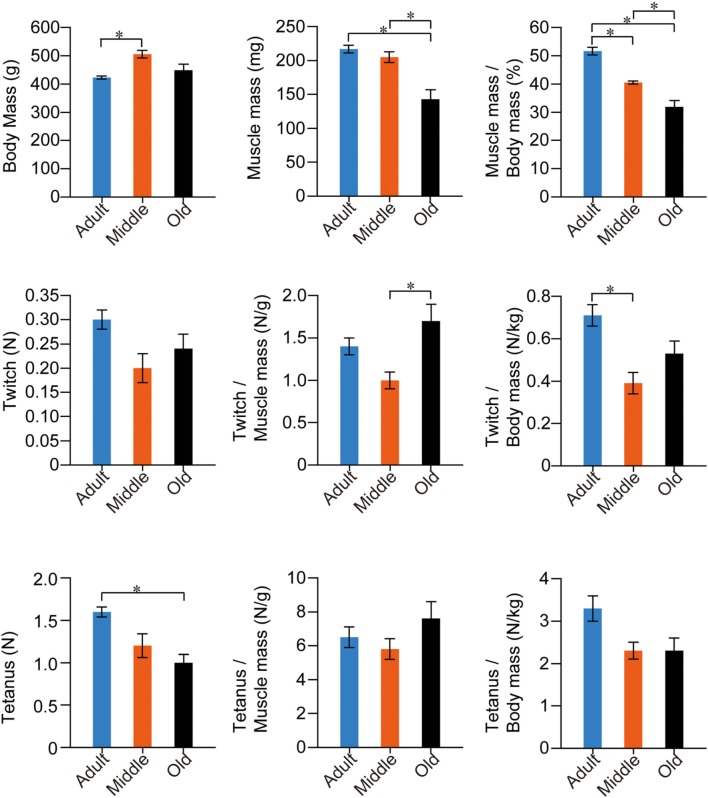
**Stats for SOL muscle functions in the three groups.** Muscle twitches and tetanus are expressed in terms of Newtons (N), and are evaluated as absolute and relative (per muscle and body mass) values. Overall significant differences among the three groups were apparently lower in slow-type SOL when compared with fast-type PLT muscles (see previous Figure 5), thus suggesting that functional decreases were more apparent in fast-type muscle with aging. ^*^p < 0.05

The stats for SOL muscle function are summarized in Figure 6. The trend was apparently different from PLT muscles. The same trend for mean body mass was observed, but significant differences were seen only between the adult and middle-aged groups. However, the adult and middle-aged groups showed similar absolute muscle mass, and the old-aged group showed a significantly lower value than the other two groups. Age dependent significant decreases were seen in muscle mass index. In functional examinations, as a whole, the adult group showed higher values than the two older groups; however, significant differences were observed only in the twitch/body mass and absolute tetanus. Interestingly, functions in the old-aged group were comparatively similar or rather higher than in the middle-aged group, both absolutely and relatively. Taken together, volumetric decreases following aging were also evident in slow-type SOL muscle, but the functional decreases were less than in the fast PLT muscle.

### Muscle shortening and relaxing velocity during twitch contraction

**Figure 7:**
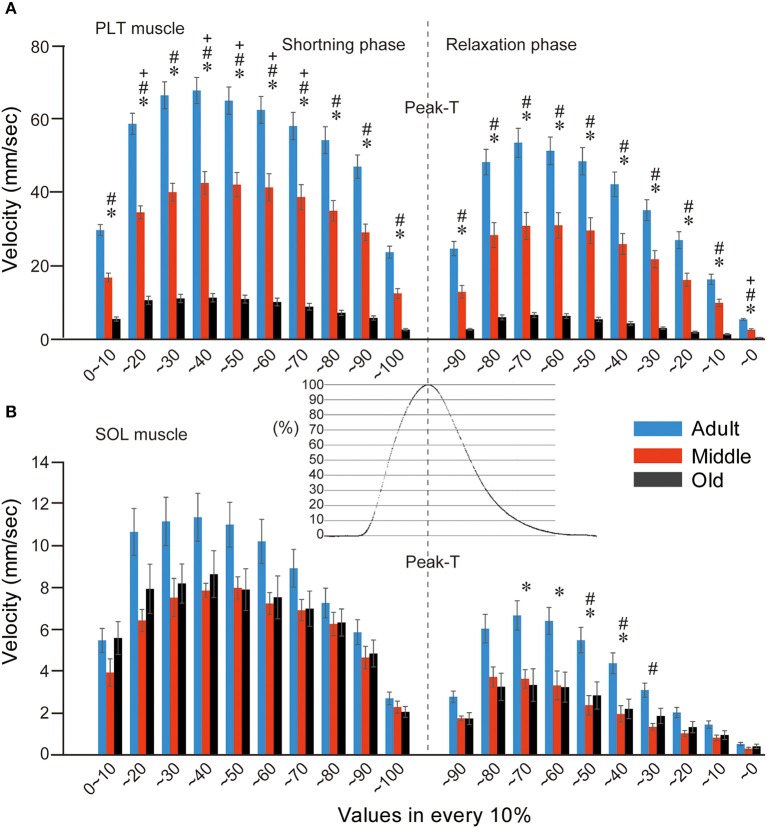
**Comparison of muscle shortening and relaxing velocity in both PLT (A) and SOL (B) muscles among adult, middle-aged, and old-aged rats.** Gradual decreases in both velocities in the three age groups were evident in the fast-type PLT muscle **(A)**. Similarly, decreases were observed between the adult and middle-aged groups, but there were no significant differences between the middle-aged and old-aged groups in slow-type SOL **(B)**. Typical raw data for twitch tension curves in PLT muscle are shown out in central area. Velocity was averaged in 10% divisions from 10 serial twitches and is presented as group values (mean's ± *SE*). ^*^P < 0.05, ^#^*P* < 0.05, ^+^*P* < 0.05.

Muscle shortening and relaxing velocity was also compared among the three groups at 10% divisions during twitch (Figure 7). In the PLT muscle, the older two groups showed significantly lower values when compared with the adult group in all division stages through the shortening and relaxing phase (Figure 7A). This group difference trend was similar to the results for tension output (as shown in Figure 5). In a comparison between the middle-aged and old-aged groups, significant decreases in the old-aged group could be seen in the 20–70% range during the shortening phase and in the 10–0% range in the relaxing phase. In this regard, it is likely that the myosin head oscillation was easily affected by aging in the fast-type PLT muscles. However, comparatively lower values were seen in the old-aged group when compared with the middle-aged throughout the shortening-relaxing phase (**A**).

In the SOL muscle (Figure 7B), comparative functional decreases were seen throughout the shortening-relaxing phase in the two older groups when compared with the adult group, but significant differences were detected during the 70–30% range in the relaxing phase (**B**), in contrast to the PLT muscle (**A**). Thus, it is likely that the aging effects are smaller in the slow-type SOL muscle than in the fast-type PLT muscle (compare **A** and **B**), and the influence was apparent in the relaxing phase, which was assumed to be due to the ATP-dependent Ca^++^ pump functions of the sarcoplasmic reticulum. However, there were no differences between the two older groups throughout the shortening-relaxing phase. These data also indicate that the functional declines in shortening-relaxing velocity of slow-type SOL muscle already occurred in the middle-aged group and did not develop until to old age.

### Evaluation of muscle fiber type distribution in adult vs. middle-aged groups

**Figure 8:**
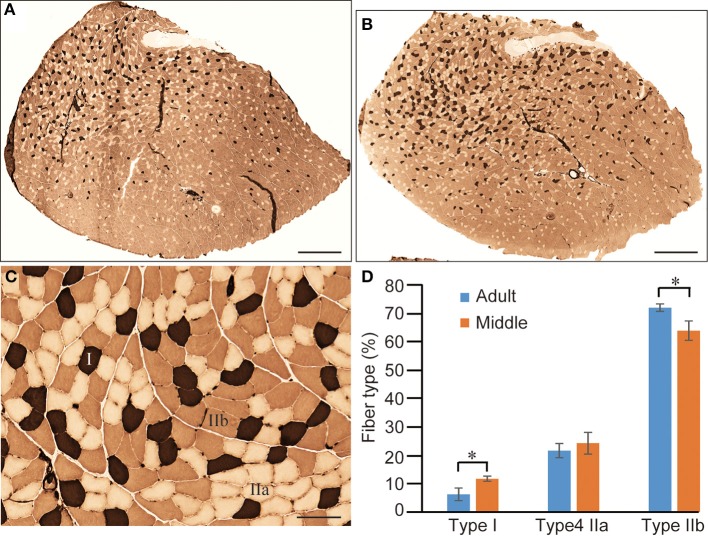
**Comparison of fiber-type components in PLT muscle between adult and middle-aged rats.** Whole PLT sections were stained for ATPase (preincubation, 4.6); thus, the darkest staining represents Type I, medium Type IIb, and light Type IIa **(C)**. This is likely to give the impression that the distribution of Type I fibers is slightly higher in middle-aged rats **(B)** than in adult rats **(A)**. Differences were significant when calculated per unit area (section) for Type I and Type IIb **(D)**, thus confirming the histochemical changes from fast- to slow-type fibers. Bars in **(A,B)** = 1 mm, **(C)** = 100 μm. ^*^P < 0.05.

With regard to the decline in shortening-relaxing velocity in the middle-aged group without an apparent decrease in muscle mass, we further examined muscle fiber type distribution in PLT muscles (Figure 8). For this analysis, the old-aged group was eliminated because they showed significant decreases in muscle mass and increases in Type I fibers with typical grouping (Figure 3), and this corresponded to the literature (Kung et al., 2014). Therefore, advanced analysis was performed between the adult and middle-aged groups. In the ATPase (pH 4.6) staining of whole cross-sections, dark-brown Type I fibers were impressively higher in middle-aged than in adult rats, whereas Type IIa (relative white in **C**) and IIb (light-brown in **C**) fibers were similar (Figures 8A vs. B). However, when these distributions were calculated, there were significant increases in Type I and decreases in Type IIb fibers, and non-significant changes in Type IIa fibers were observed in the middle-aged group (Figure 8D). Thus, the shift from Type IIb to Type I fibers appears to begin in middle-aged rats, corresponding to the results of twitch-shortening and -relaxing velocity (Figure 7A), while muscle mass was maintained (Figure 5).

### Force depressions during tetanic stimulations

**Figure 9:**
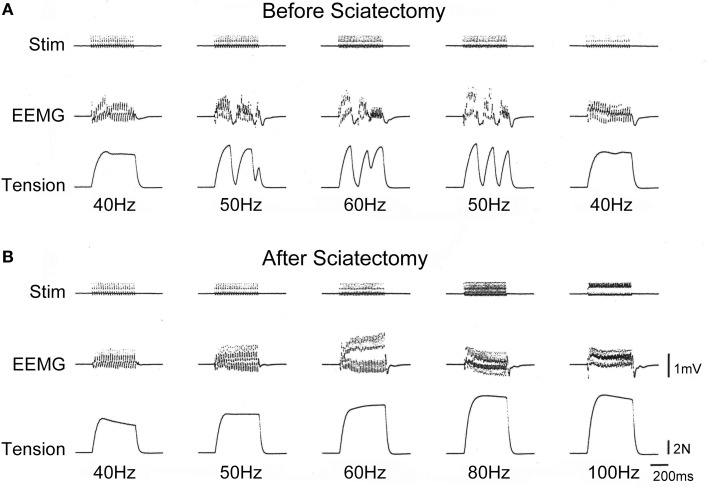
**Typical depressions of EEMG and tension force in old-aged rats *in vivo* before (A) and after (B) sciatectomy.** Sudden depression of EEMG and tension force occurred after 50-Hz stimulation, but this recovered when stimulation returned to 40 Hz **(A)**. However, there were no abnormalities observed after sciatectomy in the proximal portion from stimuli, even through 100 Hz **(B)**, thus suggesting that the cause of the decrease originated in the spinal cord. Stim, stimulation; EEMG, evoked electro-myogram; N, newton.

In addition to the declines in muscle contractile ability (as in Figures 5–7), we also detected force depressions in the older two groups during *in situ* tetanic stimulations. Typical measurement of force depressions in the old-aged rats are shown in Figure 9. Sudden force depression was detected at over 50 Hz stimulation following depression of EEMG (surface electrode), whereas normal discharges and tetanus was seen at 40 Hz (**A**). However, this trend disappeared when stimulation returned to 40 Hz. This phenomenon was confirmed in several repetitions, and means that force depressions were caused by the cut-off of electrical discharge. Subsequently, we cut the sciatic nerve (sciatectomy) at the upper portion of the stimulation site in order to confirm whether electrical depression occurred in the MN pool of the ventral horn of the spinal cord or neuromuscular junctions. We then obtained complete tetanus at 40- to 100-Hz stimulation without any force, and EEMG depression following a gradual increase in tension outputs (**B**). Therefore, this test clearly indicated that the cause of electrical cut off occurred in the spinal cord. An increase in tension output was also evident after sciatectomy (compare **A** to **B**). This also showed that recruitment of the MU, which was responsible for higher stimulation frequency, occurred after sciatectomy, and further suggested that the high threshold large MNs were affected under *in situ* physiological conditions before sciatectomy. Interestingly, the same and/or similar trends were observed in 73% (8/11) of PLT and 13% (1/8) of SOL muscles in old-aged rats, and 1/6 of middle-aged PLT and SOL muscles.

**Figure 10:**
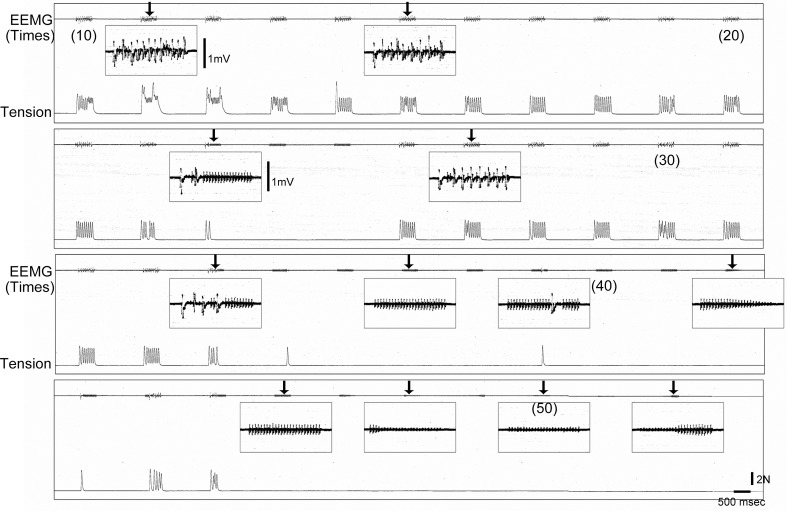
**Typical pattern of failure transmission in neuromuscular junction of old-aged rats after sciatectomy.** Gradual decrease in electrical activity associated with tension outputs could be seen following repetitive tetanic stimulation (50 Hz), and this wholly disappeared around 50 times. EEMG, evoked electro-myogram; N, newton.

Furthermore, the two above cases in the old-aged rats (2/8) continued to show abnormalities after sciatectomy. Thus, these two cases also had abnormalities in the neuromuscular junction. A typical symptom in these two cases was depressed transmission that was enhanced after fatigue. A typical pattern of abnormal neuromuscular transmission is shown in Figure 10. When tetanic stimulation was repeatedly added, the trends on EEMG and tension depression gradually became heavier following repetitions (arrows in Figure 10). Tension output appeared to completely disappear when the EEMG amplitude decreased to less than 0.4 mV, and then discharge wholly disappeared also at around 50 repetitions.

### Comparison of muscle tenderness and connective tissue content

With regard to the force depression in the older two groups during *in situ* tetanic stimulation, we believe that the muscle stiffness is closely related, and tenderness of the EDL muscle was compared among very young (age, 3-weeks; positive control), adult, and old-aged rats (Table 1). It is natural that EDL muscle length is significantly shorter in young rats when compared with the other two groups, but there were no significant differences between the adult and old-aged groups. However, significant differences were seen in both absolute and relative tenderness between all three groups. Young rats showed the highest absorption of stretching effects, even under the unfavorable conditions of this measurement, because of the shortest muscle length. In this regard, the condition of adult and old-aged rats was same, but the old-aged group showed substantial and significant losses in tenderness when compared with adults. Thus, age (sarcopenia)-dependent increases in massive muscle stiffness occurred in old-aged rats.

**Table 1:** **Comparison of muscle tenderness among 3-week-old, adult, and old-aged rats**.

	**EDL muscle length *in situ* (mm)**	**Absolute tenderness (absorbed % when stretched 6 mm)**	**Relative tenderness (absorbed % when stretched 14% of *in situ* muscle length)**
3 week-old (*n* = 4)	16.6 ± 0.3	97.9 ± 0.6	99.1 ± 0.4
Adult (*n* = 5)	38.6 ± 0.8	94.1 ± 0.9	94.7 ± 0.8
Old (*n* = 4)	42.3 ± 3.6	78.2 ± 2.1	78.2 ± 2.1
3 week vs. adult	^**^P ≤ 0.01	^**^P ≤ 0.01	^**^P ≤ 0.01
3 week vs. old	^**^P ≤ 0.01	^**^P ≤ 0.01	^**^P ≤ 0.01
Adult vs. old	ns	^**^P ≤ 0.01	^**^P ≤ 0.01

**Figure 11:**
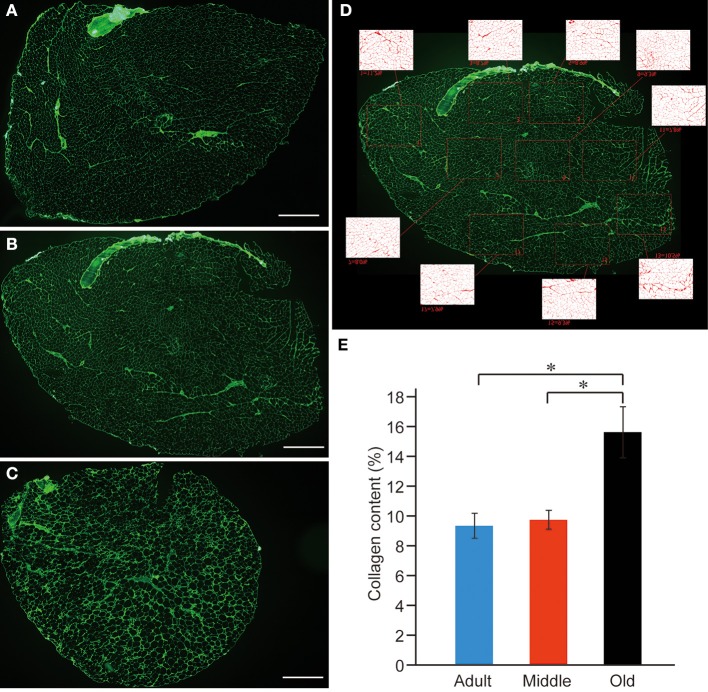
**Immunohistochemical detection of connective tissues (anti-collagen type-I) in adult (A), middle-aged (B), and old-aged (C) rat PLT muscles.** A large volume of connective tissues could be seen in old-aged rats (**A,B** vs. **C**). These were calculated in terms of percent (%) content/sections, and significant increases were evident in old-aged rats **(E)**. Panel **(D)** shows typical analysis method for middle-aged muscle. Bars in **(A–C)** = 1 mm. ^*^p < 0.05.

In order to confirm whether a similar event occurred in PLT muscles, we performed the immunohistochemical analysis of the connective tissues (collagen Type-I) in the adult, middle-aged, and old-aged groups (Figures 11A–C). As expected, the old-aged group showed significantly higher contents than the other two groups (**C** and **E**). However, there were no differences in the connective tissue contents between the adult and middle-aged groups (**B** and **E**); thus, it appears that the increase in muscle stiffness did not occur in the middle-aged group. These data suggest that muscle stiffness may also be higher in the old-aged PLT muscles, because of the increased connective tissue contents, and further suggest that this affects the functional aspects of the PLT muscle.

## Discussion

In the present study, the old-aged rats clearly showed the typical characteristics of sarcopenia, such as loss of muscle mass and strength, and slow-type fiber grouping associated with a significant reduction in the number of innervated nerve axons and myelin, specifically in fast-type PLT muscles, while the slow-type SOL muscle showed relatively mild syndromes. This is consistent with sarcopenia syndromes in humans and the experimental animals (Tomlinson and Irving, 1977; Edstrom and Larsson, 1987; Einsiedel and Luff, 1992; Deschenes, 2004). Therefore, the present old-aged rats represent a suitable sarcopenia model. In this regard, the present qualitative findings in the peripheral motor system could be also considered in the etiology and/or pathogenesis of sarcopenia: (1) reduction of shortening and relaxing velocity during serial twitch contractions; (2) decline of muscle tenderness (increased muscle stiffness following the increase in the connective tissue component); (3) impaired recruitment of MUs (sudden depression of tetanic force and EEMG) appearing during higher stimulation frequencies (over 50–60 Hz); and (4) easy fatigability in neuromuscular junctions. To our knowledge, there have been few reports describing such observations and their interrelationships in relation to sarcopenia. Concurrently, these data support several typical behaviors seen in elderly humans.

The present impaired recruitment of MUs during higher frequencies suggests the poor recruitment of high threshold fast-type MUs, and this would lead to lower muscle power output, as is observed by muscle weakness in the elderly. It is also plausible that a lower discharge rate during maximal contractions in the muscles of human lower-extremity has been reported (Kamen et al., 1995; Klass et al., 2007). Age-related decreases in MU discharge rate and force control during isometric planter flection have also been reported, even in submaximal contractions (Kallio et al., 2012). These decreases in MU discharge rate are closely related to the higher amplitude of force fluctuations in the old-age than that of the young (Enoka et al., 2003; Kallio et al., 2012). It has also been suggested that the decreased MU discharge rate is an adaptation to the increased twitch duration to optimize force generation, because twitch duration increases with age, tetanus could theoretically be achieved with lower discharge rate (Roos et al., 1997, 1999). Therefore, the present significant declines in shortening and relaxing velocity during twitch in old-aged rats (Figure 7) reasonably supported this notion.

Importantly, the present results demonstrate that the cause of these phenomena exists in the spinal cord complex, as was clearly confirmed by the higher tensions of complete tetanus at 50- to 100-Hz stimulation after sciatectomy in the old-aged rats (Figure 9). This result also demonstrated the important fact that neural alterations preceded reductions in peripheral muscle substance itself. Therefore, neural qualitative changes preceded quantitative changes following sarcopenia. We believe that the significant increase in connective tissues in the old-aged rats (Figure 11) may play a key role for the alteration of the spinal cord reflex arc. There are several possibilities regarding the related mechanisms: (1) the poor recruitment of high threshold fast-type MUs was caused by pre-synaptic inhibition in the MN pool of the ventral horn; (2) the strong firing of Golgi tendon organs (GTO) may frequently occur *in vivo* by the increase in muscle stiffness (decreased tenderness) in the old-aged rats in daily life; (3) this trend raises the sensitivity of GTO and may have resulted in facilitating the autogenetic inhibition by type Ib afferent fibers; and (4) autogenetic inhibition may be induced under the submaximal voluntary contraction (SMVC), such as the force output at 50-Hz stimulation, whereas the peripheral muscle itself retained the ability to follow 100-Hz stimuli. It is also likely that there is a mechanism for increasing the sensitivity of the recurrent inhibition of Renshaw cells, but this was not confirmed.

With the regard to ease of falling in the elderly, increased muscle stiffness through the increase in interstitial connective tissues probably also affects the sensitivity of muscle spindles. Lower sensitivity in isolated spindle afferents from the medial gastrocnemius muscle of older rats in both dynamic and static states has been reported (Miwa et al., 1995). The rationale is that increased stiffness in the spindle capsule due to the presence of more intracapsular collagen decreases dynamic sensitivity, and reduces the opening of sensory spirals. This mechanism may largely blunt the stretch-reflex *in vivo*, which is mainly related to FF-type motor units (FF-MUs), and results in insufficient exertion of instant muscle power to postural maintenance. This mechanism may also be closely related to the ability to recover from a fall, particularly in the quick response to sudden, unanticipated body imbalance, and this depends largely on maximum stepping speed (Thelen et al., 1997, 2000; Wojcik et al., 1999). For this reason, decreased recruitment and/or use of FF-MUs in daily life also facilitates the disuse depending on decreases in FF-MUs, as defined by remodeling of MU with slow-type fiber grouping in the present old-aged rats.

For the easy fatigability of neuromuscular junctions in old-aged rats (Figure 10), this may be the result of a decline in synaptic metabolic rate, because of evidence that the height of EEMG decreased and disappeared even after 20–30 repetitions of tetanus. Generally, such symptoms are not observed in normal adults over 100 repetitions. Thus, impaired uptake and/or metabolism of acetylcholine in the synaptic vesicles may be a mechanism in this case. However, this sign was observed in 2 of 8 abnormal old-aged rats; thus, it was considered to be a more progressive stage symptom of sarcopenia.

More importantly, the present study showed that a significant decrease in muscle shortening and relaxing velocity during serial twitch contractions with a shift from Type-IIb to Type-I fibers without any muscle atrophy could be detected in PLT muscle from middle-aged rats. These results indicate that qualitative decreases had begun, even in middle-aged rats, which showed no typical sarcopenia symptoms. This notion was well supported by the observation that a significant decrease in tension output both in the absolute and relative twitch and tetanus was also evident in the middle-aged rats when compared with adults (Figure 7). This state was considered to be the early stage of “dynapenia,” which consists of age-associated loss of muscle strength and power (Clark and Manini, 2012). The contributors to dynapenia are compartmentalized into two factors; (1) neurologic, and (2) skeletal muscle properties. As these factors control muscle force production (Clark and Manini, 2008; Clark, 2009), muscle size plays a relatively minor role. In this regard, the present neural and mechanical data demonstrated the potential antecedent mechanisms to dynapenia. In addition, in the analysis of shortening and relaxing velocity during twitch, a deeper influence of aging could be observed in the shortening phase of PLT and in the relaxing phase of SOL muscle. The former mainly depended on the ability of myosin head oscillation, and the latter mainly depended on ATP-dependent Ca^++^ pump functions in the sarcoplasmic reticulum (Figures 7A,B). Thus, this was also assumed to be the potential antecedent mechanisms for dynapenia on fast (PLT) and slow (SOL)-type muscles. Interestingly, similar functional decrease in the slow-type SOL muscles compared to the adult were observed both in the middle- and old-aged rats, in contrast to the age-related gradual decrease in the fast-type PLA, through the functional assessments of present study. It was supposed that this trend may represent the minimal physical/functional requirement of standard life in rats from middle- to old-aged. In other words, standard life in middle- to old-aged rats may be mainly covered by the functions of slow type MUs such as remaining in the SOL muscles. This is why; progressive decrease in the function could be seen in fast-type PLT, while the ceasing to fall at minimal requirements were observed in slow-type SOL muscle. Taken together, it is possible that dynapenia precedes sarcopenia, and/or the pathogenesis of dynapenia is the start of sarcopenia. Therefore, the present data suggests that preventing the loss of muscle tenderness and high-threshold fast-type MUs during middle age is important for the relief of subsequent sarcopenia syndrome. This notion is also supported by the observation that 1/6 of middle-aged rats already showed impaired recruitment of the MUs at higher stimulation frequency.

Considering human cases based on the present results, exercise with the preferential use of F-type (FF, FR) MUs should be performed habitually in daily life to prevent their decline of them, particularly in middle age (age, 40–60 years), that can be expecting the retain of relatively good motor abilities. This may be a good contributor to the relief of sarcopenia syndrome, particularly for falls in the elderly. Preventing the loss of muscle tenderness (ability of stretch-absorption), which is induced by increases in excess muscle connective tissues, is also an important factor, because of its influence in the spinal-cord reflex system. However, specific ideas for prevention remain uncertain in the present study, although moderate stretching of muscles with optimal nutrition may a provide foundation for prevention of muscle tenderness.

In conclusion, we demonstrated that qualitative alterations in the peripheral motor system, such as the impaired recruitment of high-threshold fast-type MUs, impaired transmission of neuromuscular junction following fatigue, loss of muscle tenderness (significantly increased muscle stiffness due to the increase in muscle connective tissues), and significant decreases of shortening-relaxing velocity during serial twitch contractions occurred as typical symptoms of dynapenia/sarcopenia syndrome. These factors may be closely related to the spinal-cord reflexes, such as the stretch and the autogenetic inhibition reflex, and decreases in these abilities can make the elderly more prone to falls.

### Conflict of interest statement

The authors declare that the research was conducted in the absence of any commercial or financial relationships that could be construed as a potential conflict of interest.

## Supplementary material

Supplementary Figure S1 **Calibration of the force-distance transducer**. High linearity was confirmed both in relation to the distance and force following quite similar equations.
