# Supplementary figures and images for: Qualitative alteration of peripheral motor system begins prior to appearance of typical sarcopenia syndrome in middle-aged rats
Source: Front Aging Neurosci. 2014 Oct 30;6:296. doi: 10.3389/fnagi.2014.00296 (PMC4214197; doi:10.3389/fnagi.2014.00296)

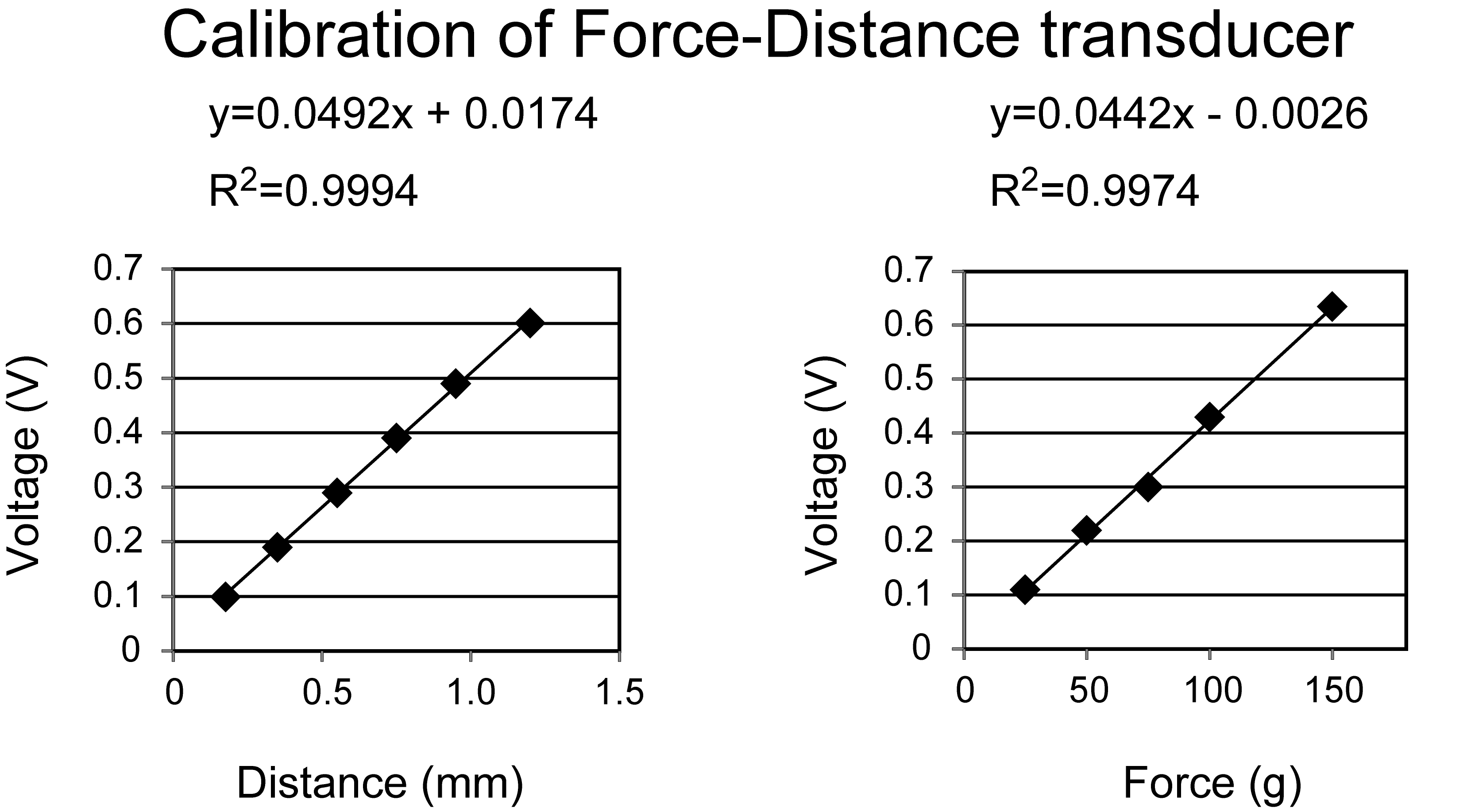

Supplement: Supplementary Figure S1 — Calibration of the force-distance transducer. High linearity was confirmed both in relation to the distance and force following quite similar equations. [file Image1.TIF]
